# Fusarium Consortium Populations Associated with Asparagus Crop in Spain and Their Role on Field Decline Syndrome

**DOI:** 10.3390/jof6040336

**Published:** 2020-12-04

**Authors:** Alexandri María Brizuela, Eduardo De la Lastra, José Ignacio Marín-Guirao, Laura Gálvez, Miguel de Cara-García, Nieves Capote, Daniel Palmero

**Affiliations:** 1 Department of Agricultural Production, Escuela Técnica Superior de Ingeniería Agronómica, Alimentaria y de Biosistemas, Universidad Politécnica de Madrid, 28040 Madrid, Spain; alexandri.brizuela@alumnos.upm.es (A.M.B.); laura.galvez@upm.es (L.G.); 2 Institute for Research and Training in Agriculture and Fisheries, IFAPA Las Torres. Ctra. Sevilla, Cazalla, Km. 12,2, Alcalá del Río, 41200 Sevilla, Spain; eduardo.lastra@juntadeandalucia.es; 3 Institute for Research and Training in Agriculture and Fisheries, IFAPA La Mojonera, Camino San Nicolás, 1. La Mojonera, 04745 Almería, Spain; josei.marin@juntadeandalucia.es (J.I.M.-G.); franciscom.cara@juntadeandalucia.es (M.d.C.-G.)

**Keywords:** Asparagus Decline Syndrome (ADS), *F. oxysporum* f. sp. *asparagi*, *F. proliferatum*, *F. redolens*, field disease index, pathogenicity, genetic diversity

## Abstract

Asparagus Decline Syndrome (ADS) is one of the main phytosanitary problems of asparagus crop worldwide. Diseased plants and soil samples from 41 fields from three main production areas of Spain were surveyed. Eight *Fusarium* species belonging to seven species complexes were identified in soils: *F. oxysporum*, *F. proliferatum*, *F. redolens*, *F. solani* *sensu stricto*, *F. equiseti*, *F. culmorum*, *F. compactum* and *F. acuminatum*. *Fusarium oxysporum* was the most prevalent species. Statistical correlation (R^2^ = 88%) was established between *F. oxysporum* inoculum density and the average temperature of the warmest month. A relationship was also established between three crop factors (average temperature, crop age and *F. oxysporum* inoculum density) and field disease indices. Significant differences were observed between the distribution of *F. oxysporum* propagules in white and green asparagus fields. Thirteen *Fusarium* species belonging to seven species complexes were identified from roots of diseased plants, being *F. oxysporum* the most prevalent. *F. proliferatum*, *F. oxysporum* and *F. redolens* showed pathogenicity to asparagus and were the main species associated to ADS. *Fusarium oxysporum* was the species with the highest genetic diversity displaying 14 sequence-based haplotypes with no geographic differentiation. This work contributes to understanding the *Fusarium* complex associated to ADS for developing accurate integrated disease management strategies.

## 1. Introduction

Asparagus (*Asparagus officinalis* L.) is one of the most widely distributed open field horticultural crops in the European Union. With more than 14,688 ha dedicated to the crop and production of 68,403 t, Spain is the fifth largest producer in the world and the second largest in Europe after Germany, both among the five biggest world producers [1]. Exports are around 24,701 t with a total value of 70.4 million euros [2]. However, the crop has experienced a substantial yield decrease over the last few seasons due to fungal diseases.

Asparagus Decline Syndrome (ADS) is one of the main phytosanitary problems of asparagus crop worldwide [3]. It is characterized by a gradual loss of vigor which can even lead to the death of affected plants. The symptoms associated with the disease are variable and can be observed in the different phases of the crop, from the seedlings and small crowns used in the plantation to the adult plants in full production. In seedling stage, the main symptomatology is root rot that, in severe cases, can cause the death of seedlings [4]. In young crowns, reddish coloration and rots in the secondary root system are observed that begin from the tips and end with the complete disappearance of the secondary root [5]. Storage roots take longer to express symptoms, and brown spots appear on them, which in their early stages do not progress in depth. Later, the roots collapse and empty, leaving only the hollow epidermal cover. In adult plants, the pathogen progresses through the vascular tissues until it reaches the crown, where a cross section of the claw allows the observation of a dark brown rot. It is in older crops where symptoms are most clearly seen. After the productive period, when the plants are left to vegetate in order to store the necessary reserves for the following year, the affected plants begin to show a premature yellowing that could be confused with that caused by root asphyxia due to waterlogging. The most affected stems become completely dry and finally the whole plant wilts. 

This is a complex syndrome in whose causality both abiotic, such as water stress or allelopathic compounds, and biotic factors could be involved [6,7], but in which the *Fusarium* complex plays a predominant role [8,9,10]. The syndrome is expressed by shortening the productive period of the crop and limiting the replanting of asparagus on soils previously cultivated with asparagus by leading to the phenomenon known as “soil fatigue” [11]. This phenomenon appears in the second and third year after replanting, which does not depend on the rest period between plantations, and is characterized by a marked decrease in the vigor of the plants combined with a smaller size of harvested sprouts. The decrease in the yield is so drastic that on many occasions it makes the crop unprofitable.

The disease was first described more than a century ago, but its biotic causal agent was not identified until 1941, when Cohen and Heald [12] described it as *F. oxysporum* f. sp. *asparagi.* Since then, different researchers have addressed the study of the disease by extending its etiology to a complex of *Fusarium* species that have been associated with the syndrome, including *F. proliferatum*, *F. redolens*, *F. solani* or *F. ershadii*, among others [9,13,14,15,16,17,18,19].

Among all of them, *F. oxysporum* f. sp. *asparagi* and *F. proliferatum* are the species that seem to prevail as the most pathogenic within the specific complex associated to the disease, although the composition of the fungal consortium is highly variable depending on the geographical region under study. ADS was first reported in Spain in the 1980s [20]. Several of the *Fusarium* species previously described in other areas of the world were also isolated in our country, particularly the most abundant species, *F. oxysporum* and *F. proliferatum* (*sensu* Messiaen and Cassini [21]), whose pathogenicity was demonstrated.

This disease is difficult to control due to the multiannual character of the asparagus crop, easy dissemination with the propagation material and the persistence of asparagus root residues and inoculum of the Fusaria consortium over time, recovering asparagus root residues with high contents of fusaric microbiota after 25 years without cultivation [9,17]. On the other hand, the available asparagus cultivars have low resistance [22,23,24]. Although there is a source of resistance to *Fusarium* in *Asparagus densiflorus* [25], it is difficult to obtain resistant cultivars under various agro-environmental conditions due to the high genetic and pathogenic diversity of the Fusaria consortium, whose species composition and preponderance varies with the climatic conditions. An accurate identification of the species associated to ADS is crucial for management. A multilocus sequence typing database, *Fusarium* MLST (http://www.westerdijkinstitute.nl/fusarium) with partial sequences from phylogenetically informative loci was developed to facilitate accurate identification of single phylogenetic *Fusarium* species. Several gene fragments can be amplified by PCR and sequenced using primers that are conserved across the phylogenetic breadth of *Fusarium*. The phylogenetic concept of species, based on DNA sequence homology and phylogenetic analysis, has allowed the description of multiple *Fusarium* species (originally associated to a single morphological species) belonging to different species complexes. In addition, estimating the genetic diversity and the population structure of *Fusarium* consortium associated to ADS in the three asparagus production areas can increase our understanding of the disease and improve the management. In this work, a multilocus sequence dataset was constructed consisting of concatenated partial sequences of genes for the translation elongation factor 1-alpha (*EF-1α*) and the DNA-directed RNA polymerase II largest (*RPB1*) and second largest subunit (*RPB2*) to accurately identify and to assess the genetic diversity of the *Fusarium* consortium associated to asparagus in Spain.

The present study aimed to describe the role of *Fusarium* consortium on ADS by analyzing asparagus fields in the main Spanish production areas. To achieve this, *Fusarium* isolates were obtained from diseased asparagus plants and soils from the north, center and south of Spain, corresponding to Navarra, Madrid and Andalusia regions. *Fusarium* isolates were morphologically and molecularly identified via multilocus sequence typing, and the relative abundance of each species in the three production areas and their pathogenicity to asparagus were determined. In addition, the potential correlation between the climatic conditions of the cultivation area and the occurrence of *Fusarium oxysporum* soil inoculum density and the relationship between three crop parameters (average climate temperature, crop age and *F. oxysporum* soil inoculum density) and field disease index (FDI) values were estimated. Finally, the phylogenetic relationships among isolates and the genetic diversity of the pathogenic *Fusarium* species detected in the Spanish production areas were calculated. 

## 2. Materials and Methods

### 2.1. Asparagus Fields Sampling

**Figure 1:**
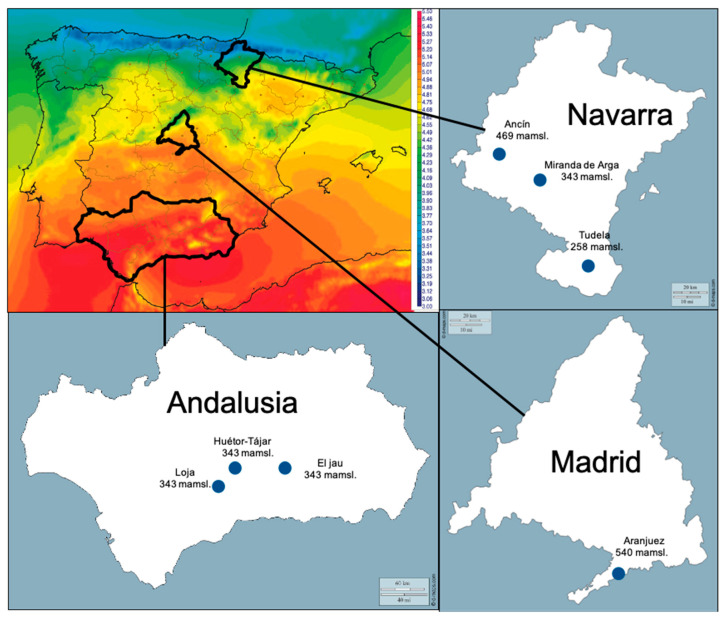
Surface Incoming Radiation map - AEMET (1983–2005) (kwh m^−2^ day^−1^) (Climate-Satellite Application Facilities), location and altitude of sampled municipalities.

Asparagus is a broad distributed crop in Spain. Sampling was designed to cover not only different cultivated varieties and growing methods but also different climatic regions. In total, 41 fields from three provinces covering seven different municipalities were sampled for soil and plants. Soils from plots with different crop ages (0–22 years) were sampled and analyzed. In total, 16 fields were collected in the north of the country (Navarra) (1–16), 6 in the center (Madrid) (17–22) and 20 in the south (Andalusia) (23–41). Sampling dates, climate and location of the sampled fields are summarized in Table 1 and Figure 1.

Sampling dates were October 2017 for fields from the north and center of the country and June and July 2018 for fields from the south. Samples from north and center were obtained during vegetative development after the harvest period. Disease Severity Index was determined in the field by two independent observers as percentage of prematurely yellowed plants. All percentage values for field disease index were arcsine square root transformed before statistical analysis. Samples from the south were collected during harvest period (Table 1). For each municipality, climatic data were collected from the regional basic climatological network.

All plant samples were manually collected and consisted in 4–6 well-developed plants per field (including the root system). Prematurely yellowed, dried and dead plants were selected for *Fusarium* spp. isolation. Within the plants, pieces of feeder and storage roots showing brown or necrotic spots, loss of feeder roots, root collapse and/or root rot were taken. Soil samples (3 L) were collected surrounding the roots at a depth of 5–30 cm from the root zone, using a disinfected drill. After collecting, samples were taken to the laboratory for analysis within 24–48 h.

### 2.2. Analysis of Plant Samples

Analysis of the plant samples consisted in the superficial disinfection of secondary and storing roots with 1.5% sodium hypochlorite solution for 1 min, followed by two successive washings with sterile distilled water. After drying, 1 cm pieces were sown in plates with potato dextrose agar (PDA) culture medium supplemented with 0.5 g/L of streptomycin sulphate (Sigma-Aldrich, St. Louis, MO, USA) (PDAS) and incubated for 5–7 days at laboratory temperature (25 °C) under continuous fluorescent light. Fungal single-spore cultures were obtained from the different *Fusarium* colonies recovered.

### 2.3. Analysis of Soil Samples

Analysis consisted of drying the soil under aseptic conditions at room temperature (20–25 °C), crushing and sifting the samples through a 200 μm sieve and adding 0.02 g of the sifted soil to a Fusarium-selective medium as described by Komada [27] modified by Tello et al. [28] (this modification contains, per L, 10 g galactose, 10 g agar, 1.25 g Pentachloronitrobenzene (PCNB) and 0.5 g streptomycine sulfate). Sixteen petri dishes per sample were used and divided into four blocks of four dishes. Plates were incubated at 25 °C under continuous fluorescent light for 10 days. The mean number of colony forming units (CFU) per petri dish and the standard deviation of the mean were calculated for all *Fusarium* species and used as the basis for comparisons. 

### 2.4. Morphological Characterization of Fungal Isolates

Morphological approach was used to assign isolates to the *Fusarium* spp. level. Procedures and taxonomic criteria of Nelson et al. [29] and Leslie and Summerell [30] were followed.

### 2.5. Molecular Characterization of Fungal Isolates

Molecular identification of representative single-spore soil and plant isolates was confirmed by sequencing of a portion of the *EF-1α* gene using primers ef1 and ef2 [31]. A multilocus sequence typing was carried out for 70 selected plant isolates: 31 *F. oxysporum*, 23 *F. proliferatum* and 16 *F. redolens* (Table 2). To do that, DNA was extracted from single-spore *Fusarium* isolates using the Isolate II Plant DNA Kit (Bioline, Toronto, Canada) following the manufacturer’s instructions. The DNA concentration was determined on an ND-1000 NanoDrop spectrophotometer (Nano-Drop Products, Wilmington, DE, USA). PCR reactions were performed to amplify and partially sequence *EF-1α RPB1* and *RPB2* genes from each selected isolate. PCR reactions contained 10× PCR Buffer (Intron Biotechnology, Inc., Seongnam, Gyeonggi, Korea), 2 mM MgCl2, 0.25 mM each dNTP, 0.4 µM each primer (Table S1), 1 U of i-*Pfu* DNA polymerase (Intron Biotechnology, Inc., Seongnam, Gyeonggi, Korea) and 20–100 ng of genomic DNA. Amplifications were carried out at 95 °C for 3 min followed by 35 cycles of 30 s at 95 °C, 20 s at 59 (for *EF-1α*), 57 (for *RPB1*) or 55 °C (for *RPB2*) and 60 s at 72 °C. The size of the amplicons was resolved in 1.5% agarose gels in 0.5× TAE (Tris-acetate-EDTA) buffer; then, they were stained with RedSafe nucleic acid staining solution (Intron Biotechnology, Inc., Seongnam, Gyeonggi, Korea) and visualized over an ultraviolet transilluminator. Amplicons were purified using the FavorPrep Gel/PCR purification kit (Favorgen, Ping-Tung, Taiwan) following the manufacturer’s instructions and sequenced by STAB VIDA DNA Sequencing Service. Sequences were deposited in GenBank and compared with available sequences in GenBank and *Fusarium*-MLST databases by BLAST analysis. The whole fungal collection was maintained on potato dextrose agar and Komada’s media and stored at 4 °C in the fungus collection of the IFAPA and in the Polytechnic University of Madrid. Representative isolates were maintained in 20% (*v*/*v*) glycerol at −80 °C. 

### 2.6. Pathogenicity Tests of Plant and Soil Isolates

In total, 37 *Fusarium* monoconidial isolates (*F. oxysporum* (*n* = 10), *F. redolens* (*n* = 8), *F. proliferatum* (*n* = 11) and *Fusarium solani* species complex (FSSC) (*n* = 8)) from diseased plants were tested for pathogenicity. For this purpose, asparagus seeds of cultivar “Grande F1” were surface disinfested by immersion with 3.5% sodium hypochlorite for 20 min and subsequently rinsed with tap water and incubated in sterile wet paper at 28 °C in the dark. Fungal isolates were cultured in Czapek–Dox broth on a rotary shaker (150 rpm) at 25 °C for 10 days. Once plants reached an appropriate size, they were inoculated by immersion of the roots in a suspension of 10^6^–10^7^ CFU/mL for 30 min. Non-inoculated control plants were root-dipped in sterile Czapek–Dox broth. The inoculated plants were transplanted into pots with 1.6 L of sterile vermiculite. Three plants were sown per pot and 12 plants for each *Fusarium* isolate. Plants were maintained in a growth chamber set at 25/18 °C (light/dark) with a 14 h photoperiod (14,000 lux) for 60 days. These experiments were carried out twice for each *Fusarium* species tested. 

On the other hand, 258 *Fusarium* isolates (*F. oxysporum* (*sensu* Leslie & Summerel, 2006) (*n* = 87), FSSC (*n* = 68), *F. equiseti* (*n* = 41), *F. acuminatum* (*n* = 32), *F. compactum* (*n* = 16) and *F. proliferatum* (*n* = 14)) from soil samples were tested for pathogenicity in a first set of pot experiments screening. This set of tests consisted of four sequential experiments. All isolates were inoculated by drenching the potting substrate (twice autoclaved vermiculite, 1 h at 120 °C each) contained in 200-mL plastic pots with 80 mL inoculum. The inocula consisted of 10^4^–10^5^ CFU/mL suspensions of each isolate in sterile water. The inoculum was prepared by grinding colonies fully covering the entire surface of PDA (*F. oxysporum*, FSSC and *F. proliferatum* isolates) or KCL-agar (*F. equiseti*, *F. compactum* and *F. acuminatum*) plates. Isolates grew at 25 °C for 10–12 days in darkness for PDA plates and for 14–15 days under UV light for KCl plates. Non-inoculated control plants were watered with an aqueous homogenize of non-colonized PDA. Seeds were disinfected as described above. Inoculations occurred when asparagus plants cv. Grande F1 had emerged on the substrate. Three plants were sown per pot and six plants were inoculated per isolate. Pots were randomly distributed and maintained for 30 days in a growth chamber under the same conditions explained above. The incidence of plants showing symptoms of infection of the roots was assessed at the end of the experiment to determine which isolates expressed pathogenicity (infection of the roots clearly higher than the control plants) for a further evaluation through a second set of inoculations.

The next set of experiments was conducted sequentially to evaluate 61 soil isolates, previously demonstrated pathogenic: *F. oxysporum* (*n* = 49) and *F. proliferatum* (12). Experiments were performed as described above for the tests of plant isolates but using 1-L plastic pots with three replicates containing seven plants each. 

Plants were evaluated weekly for disease severity based on a 1–5 scale, where 1 = 1–20%, 2 = 21–40%, 3 = 41–60%, 4 = 61–80% and 5 = 81–100% of the fronds showing chlorosis, necrosis or wilt. Area under disease progress curves (AUDPC) was calculated for each species by clustering recorded data from all plant isolates [32]. At the end of each experiment, the plants were removed from the pots, the roots rinsed in tap water, and the severity of the lesions on the root system was assessed on the same scale as fronds, related to necrosis in the root system. The fronds and root wet weights were also measured. Mean ± standard error of root rot severity and fronds severity ratings and weight loss with respect to the control, over 12 replicate plants inoculated with each isolate, were determined. The proportion of plants that were dead was also assessed at the end of the experiments. All the dead plants and several randomly assigned symptomatic plants per isolate were analyzed for re-isolation on PDA. 

### 2.7. Phylogenetic Analysis 

Phylogenetic analyses of the 70 plant isolates of *F. oxysporum*, *F. proliferatum* and *F. redolens* species were carried out by the analysis of three phylogenetically informative loci: the translation elongation factor-1α (*EF-1α*) and the DNA-directed RNA polymerase II largest (*RPB1*) and second largest subunits (*RPB2*). These loci were chosen due to be the most informative for species-level identifications and to be well represented in the database [33,34,35,36]. These sequence fragments were manually concatenated (2 598 nucleotides). In addition, sequences from *Fusarium oxysporum* species complex (FOSC), *Fusarium fujikuroi* species complex (FFSC) and *Fusarium redolens* species complex (FRSC) retrieved from GenBank were included in the analyses. For the analysis of FOSC, some GenBank isolates were used belonging to Clades 1–4 defined by O’Donnell et al. [37] and later grouped into PS1 and PS2 phylogenetic species according to Laureance et al. [38]. Multiple sequence alignments were performed in MEGA7 software using the CLUSTALW algorithm refined with MUSCLE and edited manually. Phylogenetic analyses were conducted using MEGA7 through maximum likelihood (ML) analysis using the kimura-2 parameter model for the analysis of FOSC, FFSC and FRSC isolates independently, all of them with a rate of variation across sites [39]. Support for internal branches was assessed by 1000 ML bootstrapped pseudo replicates of data. Nodes with bootstrap support ≥ 70% were indicated in the final trees. 

### 2.8. Genetic Diversity 

Haplotype distribution of the asparagus isolates belonging to FOSC, FFSC and FRSC was performed using DNA sequence information from concatenated loci by DnaSP v5 software [40]. Number of haplotypes (h), haplotype (gene) diversity (H) and nucleotide diversity (π) of the species complexes were calculated by DnaSP v5. *Fusarium oxysporum* plant isolates were artificially grouped into three populations corresponding to the three prospected production areas to discern potential differences among populations. Chi square statistic [41] was used to calculate genetic differentiation among populations. 

### 2.9. Statistical Analysis of Data

Analysis of variance on the *Fusarium* isolates from soil at different crop ages (0–22 years), average temperature of the warmest month, altitude (meters over the sea level) and type of consumption (white asparagus vs. green asparagus), as well as for *Fusarium* spp. isolated from feeder and storage roots and those used for pathogenicity test evaluation (fronds and root weights), were performed using Fisher’s least significant difference (LSD) tests at 99.9% confidence were carried out using STATGRAPHICS Centurion XVIII statistical package software (StatPoint, Inc., Herndon, VA, USA). Additional non-parametric Kolmogorov–Smirnov, Mann–Whitney–Wilcoxon and Kruskal–Wallis tests were performed when the assumption of the normality of the distributions for the two samples studied was not sustainable (disease severity on fronds). Differences among pathogenicity trials were not found (*p* ≥ 0.05), thus data were clustered for consistency of analysis. Arcsine square root transformation was applied to weight loss percentages. 

To determine direct correlation between the density of inoculum of any of the *Fusarium* species and the Field Disease Index (after arcsine square root transformation), simple regression analysis was adjusted to the non-linear Y-square model as it showed the highest R^2^ value. The same statistical package software was used for the generalized linear model (Y_i_ = *β*_0_ + *β*_1_X_1,i_ + *β*_2_X_2,i_ + *β*_3_X_3,i_ + … + *β*_k_X_k,i_ + ε_i_), where “Y” is the response variable (Field Disease Index) “ß_1_X_k,i_” the predictor variables and “i” the error. To determine when a pair of variables was effectively correlated, the *p*-value of its correlation coefficient was calculated (correlation coefficient test). If the *p*-value is less than or equal to 0.05, the linear correlation of the two variables is statistically significant at 5% confidence level. 

## 3. Results 

### 3.1. Characterization of Fusarium Soil Communities and Correlation with Climatic Features

**Figure 2:**
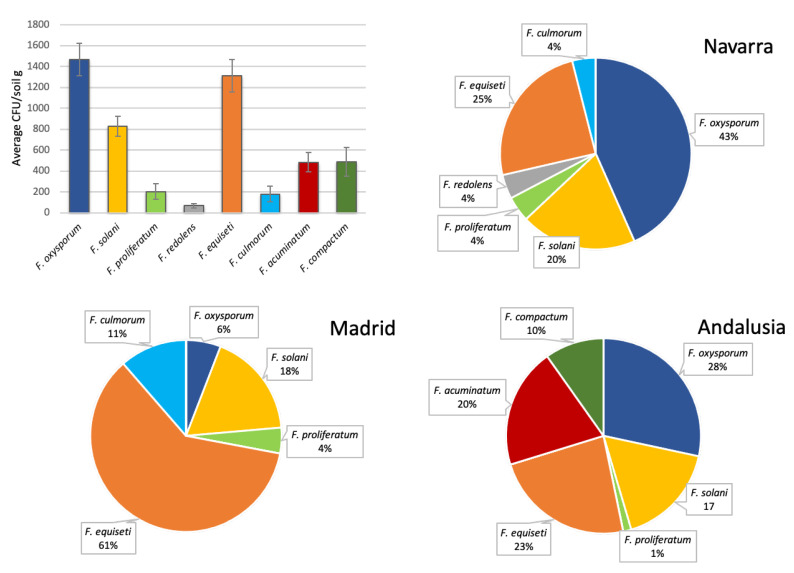
Average values of CFU/g of soil for the different *Fusarium* spp. and relative frequencies of isolation of *Fusarium* soil communities in the three analyzed production regions.

Eight species belonging to seven species complexes of the genus *Fusarium* were identified from the analyzed soil samples: *F. oxysporum* from FOSC (*F. oxysporum* species complex), *F. proliferatum* from FFSC (*F. fujikuroi* species complex), *F. redolens* from FRSC (*F. redolens* species complex), *F. solani sensu stricto* from FSSC (*F. solani* species complex), *F. equiseti* from FIESC (*F. incarnatum-equiseti* species complex), *F. culmorum* and *F. compactum* from FSAMSC (*F. sambicinum* species complex) and *F. acuminatum* from FTSC (*F. tricinctum* species complex). All three provinces analyzed showed differences in the relative frequencies of isolation of each species, although the two areas with the longest history of cultivation, Navarra and Andalusia, showed similar patterns of distribution of the main species isolated (Figure 2). 

The predominant species in the fields of both provinces was *F. oxysporum*, belonging to FOSC. This species presents the highest percentages of colonies per gram of soil analyzed (Figure 2), with average values of 2893 ± 1935 CFU/g in Navarra and 1126 ± 933 CFU/g in Andalusia. The second specific group in importance in both provinces is FIESC, followed by the isolates belonging to FSSC. The distribution in the region of Madrid varies considerably. The isolates belonging to the FIESC appear in a greater number in the soil samples of Madrid than in the other two regions, with mean values of 3240 ± 1343 CFU/g, much higher than the inoculum density of FSSC isolates and almost ten times higher than the values of the FOSC isolates.

*F. proliferatum* was isolated in similar amounts in the three sampled regions. *F. redolens* was isolated from soil samples of Navarra with an average of 287 ± 364 CFU/g of soil and from soils of Andalusia (with a density of inoculum not determined). *F. acuminatum* and *F. compactum* were isolated only from Andalusia. *F. culmorum* was isolated from the northern and central zones. 

The age of the crop had no statistically significant effects on the total density of *Fusarium* inoculum (UFC/g of soil) (*p* = 0.090). No direct correlation was detected between the density of inoculum of any of the species and the Field Disease Index (FDI) values observed during the initial sampling in Madrid and Navarra fields except for *F. oxysporum*. The simple regression analysis showed *p*-value = 0.034 for *F. oxysporum*, so the linear correlation with FDI is statistically significant at 5% confidence level. *F. oxysporum* colonies showed the highest correlation with R^2^ value of 10.25% (Table 3).

**Figure 3:**
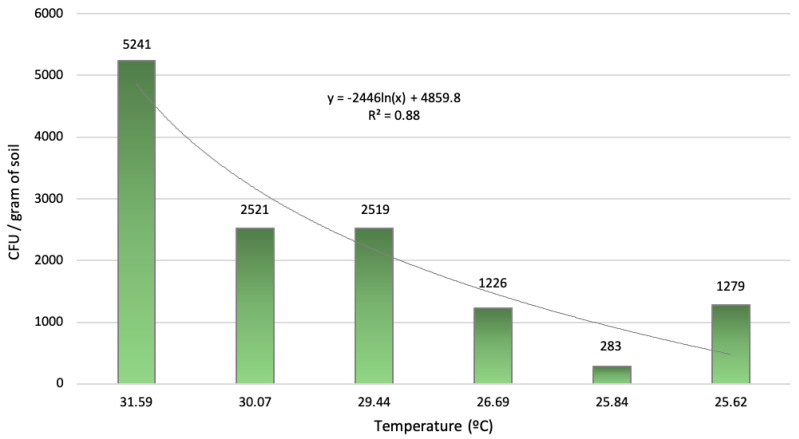
*Fusarium oxysporum* soil inoculum density isolated from sampled fields depending on the average temperature of the warmest month.

A high correlation (R^2^ = 88%) was detected between *F. oxysporum* inoculum density and the average temperature of the warmest month of the year (Figure 3). The results indicate that the *F. oxysporum* inoculum increases significantly as temperature does, coinciding with recent works where the abundance of soil pathogens was directly linked to temperature [42] (Delgado-Baquerizo et al., 2020). 

The GLM method allowed estimating the repeatability and reproducibility of the FDI measurement R-Squared = 59.27% (Adjusted R-Squared = 53.10%). After the stepwise variable selection, five effects were selected in the model, including year of cultivation, inoculum density (or CFU/g of soil) and second-order interactions (Field Disease Index = −4,6-(0.077917 · CFU) + (0.585313 · T^re)^ + (1.52425 ·Years) + (0.00245418 · CFU · T^re)^ + (0.00130563 · CFU · Years).

**Figure 4:**
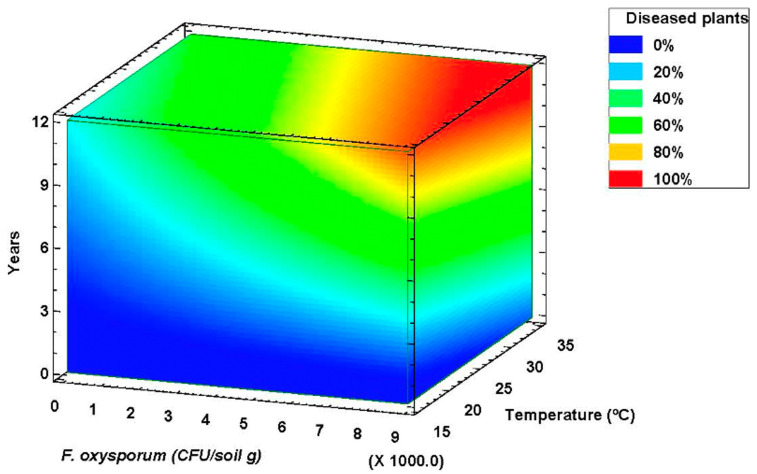
Disease Severity Index response surface contour plot at varying *Fusarium oxysporum* soil inoculum density, average temperature of the warmest month and number of years after asparagus plantation.

The fact that this is a multiannual crop has made it possible to include the age of the crop in the model. The difficulty of modeling a disease caused by a soil fungus is evident, but contour plots have proved useful for establishing the response values. The 3D surface contour plot shown in Figure 4 allows understanding the relationship between the three main factors (average temperature, crop age and inoculum density) and the FDI response values. All three variables affect FDI values of affected fields. The dark blue region identifies the lowest percent FDI, and it decreases as the amount of inoculum in the soil increases. Temperature has an effect, but a smaller one: FDI increased rapidly within the temperature, although with moderate or low inoculum levels this effect does not translate into field damage. The contour levels reveal a peak of affected plants (%) in fields with more than eight years and 6000 CFU/g of soil of *F. oxysporum*. FDI scores in this peak region are greater than 90%. At that soil inoculum levels, intermediate FDI values can be observed at all temperature regimes in fields between three and seven years age (Figure 4). 

**Figure 5:**
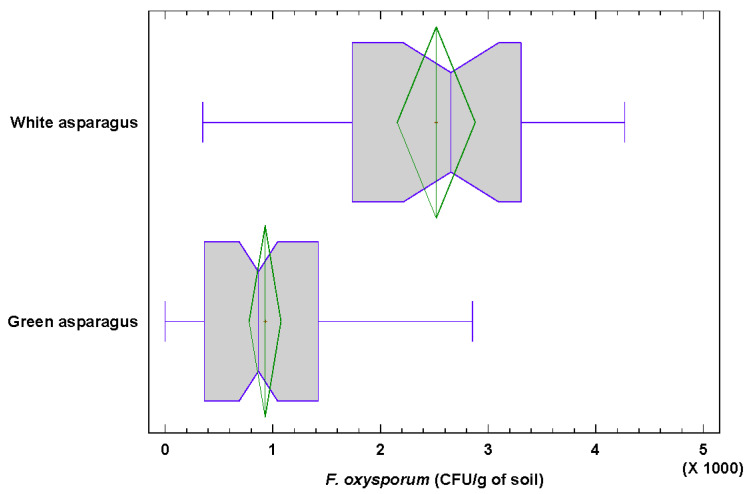
Boxplots showing mean CFU/g of soil depending on the cultivation type. Right of the box is the 75th percentile, the left is the 25th percentile and the whiskers represent the maximum and minimum values.

The number of *F. oxysporum* colonies per gram of soil isolated from fields dedicated to white and green asparagus was also analyzed. The comparative study allowed determining statistically significant differences (*p* = 2.57 × 10^−8^) between both asparagus types. The distributions of the CFU/g of soil of *F. oxysporum*/g are displayed in the boxplots below (Figure 5). The number of CFU/g soil isolated from green asparagus fields varies much less than that of white ones. 

The average values of soil were 2860 ± 1649 CFU/g for white asparagus fields and 1105 ± 1082 CFU/g for green asparagus ones. The 25% (Q3) highest CFU/g of soil in white asparagus fields were all higher than the maximum level for green asparagus fields.

The maximum distance obtained in the Kolmogorov–Smirnov test, denoted by DN, is equal to 0.622 for the CFU/g of soil data. The *p*-value is less than 0.05 (*p* = 2.57 × 10^−8^), so there is a significant difference between the CFU distributions of *F. oxysporum* in white and green asparagus fields at 5% significance level.

### 3.2. Characterization of Fusarium Plant Communities

**Figure 6:**
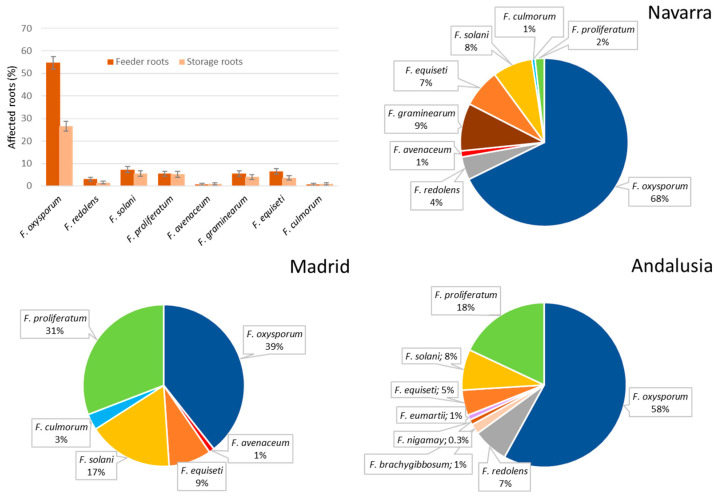
Average values of isolation from asparagus plants for the different *Fusarium* spp. depending on the root type and relative frequencies of isolation of *Fusarium* communities in the three analyzed production regions.

In total, 430 *Fusarium* isolates were obtained from 215 affected plants analyzed. Thirteen species were identified from the root system of the affected plants belonging to seven species complexes: *F. oxysporum* (FOSC)*; F. proliferatum* and *F. nygamai* (FFSC)*; F. redolens* (FRSC), *F. solani sensu stricto*, *F. falciforme*, *F. tonkinense and F. eumartii* (FSSC); *F. culmorum*, *F. brachygibossum and F. graminearum* (FSAMSC); *F. avenaceum* (FTSC); and *F. equiseti* (FIESC). *F. oxysporum* was the most prevalent species isolated from diseased plants roots, and the rest of *Fusarium* species detected showed low percentages of isolation (Figure 6). The isolation percentages of the different species vary according to the type of root (feeder or storage root), although only the isolation percentages of *F. oxysporum* showed statistically significant differences (*p =* 0.000) between the type of root analyzed. Isolation rate of *F. oxysporum* from feeder roots was 54.74%, and 26.60% from storage roots. 

### 3.3. Pathogenicity of Fusarium Species Associated to Asparagus

The pathogenicity tests revealed that 90% of *F. oxysporum*, 87.5% of *F. redolens* and 90.9% of *F. proliferatum* plant isolates were pathogenic to asparagus, while a first screening revealed that 64.4% of the *F. oxysporum* and 92.9% of the *F. proliferatum* soil isolates were pathogenic to asparagus. None of the FSSC, *F. equiseti*, *F. compactum* and *F. acuminatum* isolates expressed any symptom on asparagus seedlings (Table 4). The isolates inoculated were re-isolated from inoculated plant tissues. 

In general, *F. proliferatum* isolates produced the most severe symptoms, followed by *F. oxysporum* and *F. redolens* (Table 5). However, aggressiveness was not uniform among isolates belonging to the same species. The distribution of isolates by their aggressiveness based on plant mortality showed that *F. proliferatum* was the most aggressive species. 

**Figure 7:**
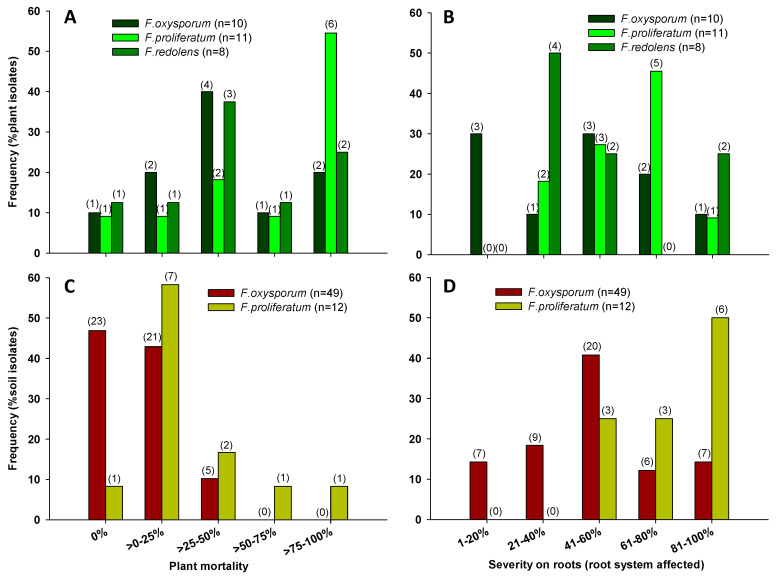
Distribution of plant and soil isolates of the pathogenic *Fusarium* spp. according to: their mortality to asparagus, respectively (**A**,**C**); and their severity on roots, respectively (**B**,**D**). Values in brackets represent the number of isolates.

In the case of plant isolates, for 54.5% of *F. proliferatum* isolates, more than 75% of inoculated plants died at the end of the experiment, while this high mortality was induced by only 20% of *F. oxysporum* isolates and 25% of *F. redolens* isolates (Figure 7A). In addition, all *F. proliferatum*, *F. oxysporum* and *F. redolens* isolates produced damages on the asparagus root system. Over 50% of *F. proliferatum* isolates produced more than 60% of rot roots, while only 30% of *F. oxysporum* and 25% of *F. redolens* isolates were so aggressive (Figure 7B). In the case of soil isolates, 16.7% of *F. proliferatum* isolates killed more than 50% of inoculated plants, while none of the *F. oxysporum* isolates induced mortality to more than 50% of plants, and 46.9% of *F. oxysporum* isolates did not kill any plants at the end of the tests (Figure 7C). However, all the isolates produced damages on the asparagus root system. For this symptom, 14.2% of *F. oxysporum* and 50% of *F. proliferatum* isolates were highly pathogenic, rotting more than 80% of the root system. The distribution of isolates by their aggressiveness based on root impact was different for both species; all *F. proliferatum* isolates were moderate to highly pathogenic, while *F. oxysporum* isolates ranged across all degrees of damages in a normal distribution (Figure 7D). This fact reveals the diversity of *F. oxysporum* found in soils concerning pathogenicity, a reflection of the versatility and biological heterogeneity for this species [43] (Gordon and Martyn, 1997). *F. proliferatum* was pathogenic for all cases, even though the studied isolates came from soils, not from plant tissues.

With regards to the effect of the inoculations on fronds and roots weights, root damages can explain the decrease of weights for all the species assessed. *F. proliferatum* produced more damages on roots and reduced asparagus biomass production, in a higher extent than *F. oxysporum* and *F. redolens*. Relative root weights decreases were higher than fronds decrease for the three species. The highest impact was observed for *F. proliferatum*, regardless the origin of the isolates (Table 5).

**Figure 8:**
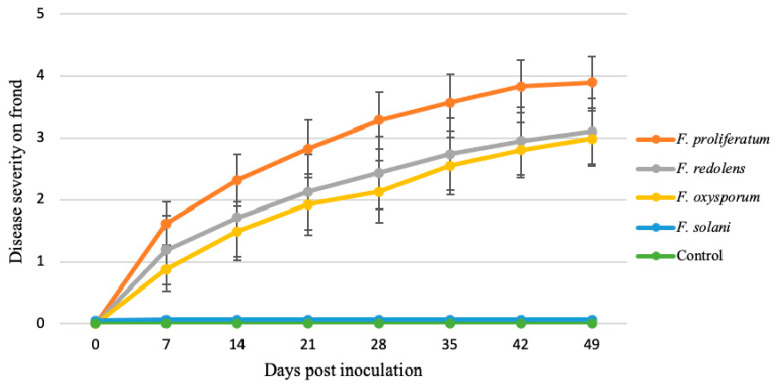
Progress of the severity of symptoms in “Grande F1” asparagus plants inoculated with *Fusarium* spp. Severity of plant symptoms was measured weekly from one to seven weeks after inoculation on a 0–5 rating scale. Values are means ± standard error over the number of isolates for each species. Non-inoculated plants were used as control.

In addition, *F. proliferatum* plant isolates presented AUDPC values significantly (*p* = 0.000) higher than the exhibited by *F. oxysporum* and *F. redolens* isolates, and these values were significantly higher than those presented by FSSC isolates and the non-inoculated control, which did not present any aerial symptoms (Figure 8).

### 3.4. Phylogenetic Analysis 

**Figure 9:**
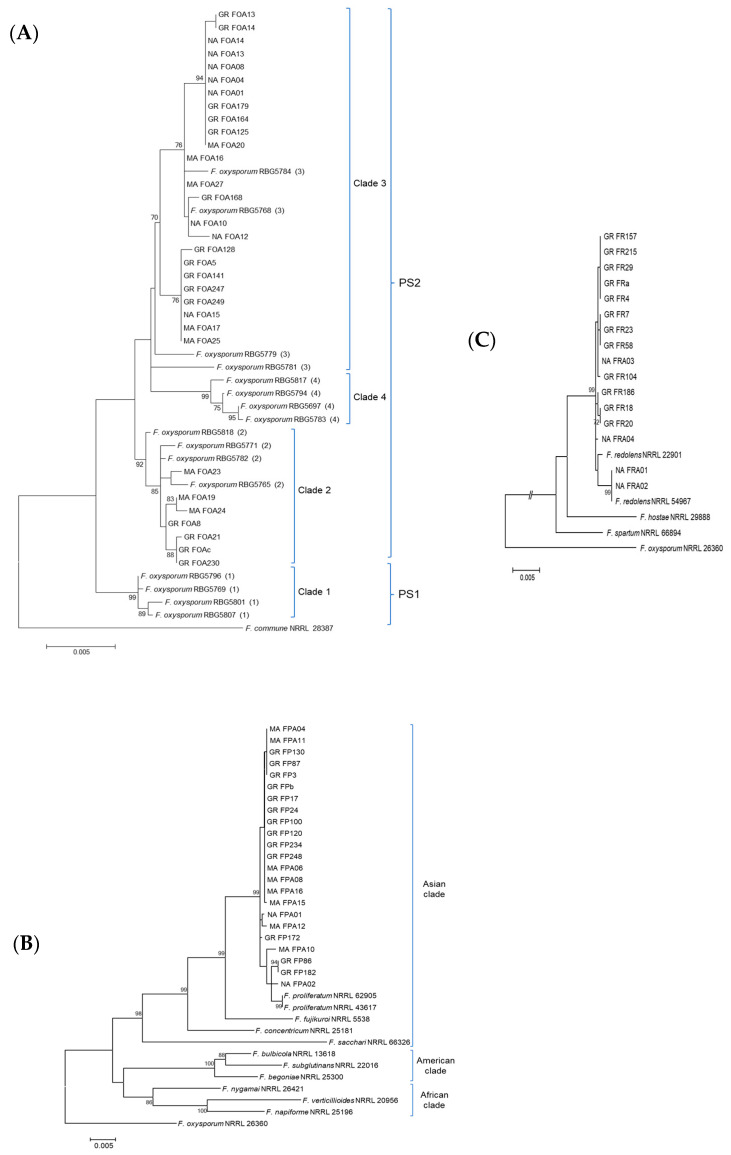
Phylogenetic diversity of *Fusarium oxysporum* (**A**), *F. proliferatum* (**B**) and *F. redolens* (**C**) isolates from asparagus diseased plants of Navarra (NA), Madrid (MA) and Andalusia (GR) production fields, using maximum likelihood analysis of the concatenated translation elongation factor-1a (*EF-1α*) (704 base pairs), DNA-directed RNA polymerase II largest (*RPB1*) (1810 base pairs) and second largest subunit (*RPB2*) (1596 base pairs) sequence data. *Fusarium commune* NRRL 28387 and *F. oxysporum* NRRL26360 were used as outgroups. Support values are above branches and represent bootstrap values of > 70%. For *F. oxysporum*, the clade designation of O’Donnell et al. (2004) is indicated in parenthesis for isolates retrieved from GenBank, and the phylogenetic species 1 (PS1) and 2 (PS2) correspond to the designation of Laurence et al. (2014). For *F. proliferatum*, the three clades established by O’Donnell et al. [31] are indicated.

*F. oxysporum* isolates associated to asparagus diseased plants grouped with isolates from Clades 2 and 3 of FOSC and were classified into phylogenetic species 2 (PS2) according to Laurence et al. [38] with high bootstraps support. Clade 3 was the most numerous group and included isolates from the three regions analyzed. Clade 2 contained isolates from Madrid and Andalusia (Figure 9A). The phylogenetic analysis of FFSC isolates from asparagus plants identified all the analyzed isolates as *F. proliferatum*. They grouped with *F. fujikuroi*, *F. concentricum* and *F. sacchari* into a well-supported group defined as the “Asian Clade” by Kvas et al. [44], and significantly differ from species belonging to the American and African Clade (Figure 9B). *F. redolens* was the only species of the FRSC detected in asparagus diseased plants. In the phylogenetic analysis, all asparagus isolates grouped with *F. redolens* isolates retrieved from the GenBank and significantly differ from *F. hostae* and *F. spartum* species with high bootstrap support (Figure 9C).

### 3.5. Genetic Diversity 

**Figure 10:**
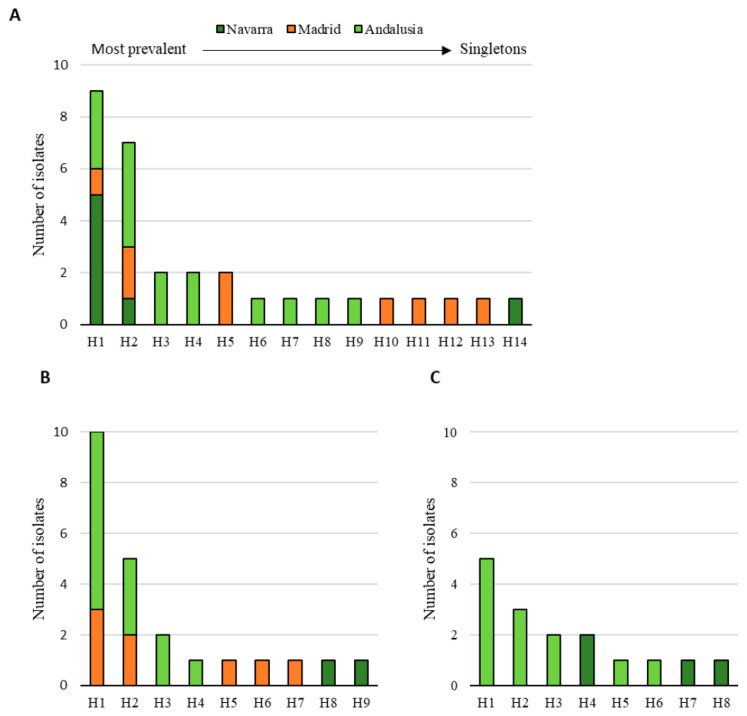
Histogram showing the distribution of isolates from diseased plants from asparagus production fields located in Navarra, Madrid and Andalusia, defined as number of isolates per multilocus haplotype: *Fusarium oxysporum* (**A**); *F. proliferatum* (**B**); and *F. redolens* (**C**).

Haplotype analysis of the pathogenic *Fusarium* species associated to asparagus diseased plants showed that *F. oxysporum* was distributed among 14 haplotypes, and *F. proliferatum* and *F. redolens* presented nine and eight haplotypes, respectively. The most prevalent haplotypes were present in *F. proliferatum* and *F. oxysporum* species. Some *F. oxysporum* isolates from Navarra, Madrid and Andalusia shared the same haplotype (H1 and H2), as did some *F. proliferatum* isolates from Madrid and Andalusia. The distribution of haplotypes was strongly tailed in all analyzed *Fusarium* species. Unique multilocus haplotypes (singletons) were observed in all species complexes and in the three regions, except for *F. redolens* that was not detected in plants from prospected fields in Madrid (Figure 10). 

Genetic diversity analysis showed that *F. oxysporum* was the species with the highest genetic (haplotypic and nucleotide) diversity. *F. proliferatum* presented the lowest haplotypic diversity and *F. redolens* the lowest nucleotide diversity (Table 6). Measures of genetic differentiation were performed for *F. oxysporum* plant isolates artificially grouped into three populations corresponding to the three prospected areas, to discern differences between populations related to the geographical origin of isolates. No differentiation (*p* value of X^2^ = 0.0900) was detected between the *F. oxysporum* populations of Navarra, Madrid and Andalusia production regions (Table S2). 

## 4. Discussion 

The main objective of this work was to discern the role of the *Fusarium* consortium on the Asparagus Decline Syndrome, which severely compromises the current asparagus production in Spain. To achieve this, diseased asparagus plants and soil samples were collected from asparagus fields located in the three main production regions of Spain and studied. 

Navarra and Andalusia regions, located in the north and south of the country, respectively, have a long history of asparagus cultivation, and both areas presented a similar pattern of distribution of the main *Fusarium* species detected in the soil, with the prevalence of *F. oxysporum*. Analysis of the plant crowns and pathogenicity tests confirmed that the fungus is capable of colonizing the vascular system of the plants, indicating that *F. oxysporum* f. sp. *asparagi* is present in diseased plants, as well as in soils, where this *forma specialis* coexists with *F. oxysporum* isolates non-pathogenic to asparagus. 

This similar pattern of *Fusarium* species distribution is maintained despite the type of cultivation and asparagus variety used, which is very different between the two areas: while in Andalusia the asparagus is harvested green, when the shoot emerges from the ground and reaches a height of about 20 cm, in Navarra, the plants are grown deeper and under a black plastic cover to prevent the shoot from synthesizing chlorophyll. In this region, the quality of the asparagus is linked to the white color of the fronds, and, for that reason, nighttime harvesting is carried out. Plastic cover allows maintaining the humidity of the soil for much longer, avoiding evapotranspiration and raising the surface temperature, so that the way of cultivation could be favoring the multiplication of the fungus in the soil. Fungal inoculum remains at upper levels in fields dedicated to white asparagus but is more variable, especially at low inoculum level. 

Madrid, the central region of asparagus production, presented a different pattern of species distribution probably due to the most recent history of cultivation of this region. The low number of years that asparagus has been grown in the area has not allowed the microbiota associated with the crop to evolve into specialization in the same way as in the other two sampled regions. This can be in concordance with Blok and Bollen [15], who found a negative relationship between the number of asparagus-free years and the inoculum density in soils.

A high correlation (R^2^ = 88%) was established between *F. oxysporum* inoculum density in the soil (CFU/g of soil) and the average temperature of the warmest month of the year. In recent years, the disease has spread through many production areas, warming at the global scale has brought about an evolution of the pathosystems. Recent studies have shown a direct association between mean annual temperature and the abundance of groups of soil-borne fungi, some of them plant pathogens [42]. In the specific case of the asparagus vascular wilt, the warming at the global scale could compromise the crop in many production areas.

The prevalence of *F. oxysporum* compared to other species isolated from asparagus roots (with isolation percentages between 7 and 90 times higher) allows us to assume the greater importance of this species in the early stages of colonization of asparagus plants via the root system. In addition, a positive correlation was found between *F. oxysporum* f. sp. *asparagi* inoculum density in the soil and FDI, indicating that the incidence of dead plants was related to the quantity of initial inoculum. In addition, the age of the culture and the average temperature also affects the infection of plants in the field. On the other hand, the greater number of feeder roots affected by *F. oxysporum* compared to storage roots, pointed to feeder roots as the first point of entry. The propagules would germinate and colonize them to later pass to the reserve roots. The main point of entry of the pathogen into the plant is through cuts made to the asparagus shoots during harvest, but entry through the root system should not be underestimated. In this sense, planting soil could be suggested as a source of inoculum for pathogenic *Fusarium* species. The fact that *F. oxysporum* was the most prevalent species detected in the soil and in diseased asparagus plants, strongly supports this hypothesis. For that reason, strategies to reduce the level of *Fusarium* inoculum in pre-planting production soils, such as incorporating organic amendments in biosolarization or biological control [45,46], are highly recommended. 

With regard to the pathogenicity of *Fusarium* species assessed, the *Fusarium* species found pathogenic (*F. oxysporum*, *F. proliferatum* and *F. redolens*) do not differ from those associated with ADS in previous works [9,16,17,18,19]. However, it differs from the last information concerning the Spanish asparagus fields sampled 15 years ago, where *F. solani* was shown as a very pathogenic species [17]. Seventy-six FSSC isolates were tested in the present work, and none of them was pathogenic to asparagus. FSSC, currently defined as genus *Neocosmospora* [47], includes many species that were previously considered *F. solani* (section Martiella & Ventricosum, Nelson et al. [29]). This can explain to some extent our differential results. Probably, FSSC species detected in this study (*F. solani sensu stricto*, *F. falciforme*, *F. tonkinense* and *F. eumartii*) were different from the former *F. solani* associated to asparagus in the past. Obviously, we did not find those pathogenic *F. solani* isolates in our recent survey. Similar explanation can be found for the works of Lamondia and Elmer [48] and Schreuder et al. [14], who did not find pathogenicity for *F. solani* isolates associated to ADS plants.

*F. proliferatum* was the most aggressive pathogen of the consortium. Even though *F. proliferatum* does not produce chlamydospores, thus its survival in soils is supposed to be lower than *F. oxysporum* or *F. redolens*, the pathogenic ability of *F. proliferatum* showed on roots was higher than the other species, causing severe necrosis and death of plants. These observations are in concordance with those of Block and Bollen [49] and Tello et al. [20]. *F. oxysporum*, *F. proliferatum* and *F. solani* have been associated to garlic crop in Spain, in cropping areas nearby the asparagus fields [50], which could be linked with the presence of *F. proliferatum* in the environment, as well as the prevalence of pathogenic isolates of the chlamydospore-forming species (*F. oxysporum*, *F. solani*) in the soils. The pathogenicity of *F. oxysporum* f. sp. *asparagi* and *F. redolens* was quite similar: both species have common morphological features, and both produce the same type of spores, so their identification might be quite complicated. A precise molecular identification based on multilocus sequence typing via *Fusarium* MLST and phylogenetic analysis allowed the accurate identification of the species associated to ADS, which is crucial for management.

In addition to being the most abundant species detected in asparagus soils and diseased roots, *F. oxysporum* exhibited the highest genetic diversity compared to the other two pathogenic species, *F. proliferatum* and *F. redolens*, displaying the highest number of haplotypes (gene diversity) and highest number of DNA polymorphisms (nucleotide diversity). This high genetic diversity has been previously observed for this species within FOSC [51] and could imply a mayor difficulty for its control. The ability of this fungus to survive in plant debris and soil for long periods makes its control a big concern, especially when chemical and biological control has proven to be ineffective with high inoculum density in the soil [52].

No genetic differentiation was detected between *F. oxysporum* f. sp. *asparagi* isolates in the three asparagus production regions analyzed. In fact, isolates from the three regions grouped in the same phylogenetic group and even shared the same haplotype. This indicates that geographical origin was not strongly correlated with isolate grouping, despite the different history of cultivation and the use of different varieties and culture management in the three Spanish production regions.

## 5. Conclusions

Although eight and thirteen *Fusarium* species were, respectively, associated to ADS affected asparagus fields and symptomatic roots, our results suggest that the three most pathogenic species in Spanish asparagus fields turned are *F. oxysporum* f. sp. *asparagi*, *F. proliferatum* and *F. redolens*. *F. oxysporum* was the most prevalent species in all the sampled areas and *F. proliferatum* the species whose isolates showed the greatest pathogenicity to asparagus.

This work has also revealed a high genetic diversity of *F. oxysporum* species compared to the other two pathogenic species, although no genetic differentiation related to geographical distribution could be detected.

Our results also reveal epidemiological information about *F. oxysporum* in asparagus fields, describing the effects of ecological factors such as the temperature on the density of *F. oxysporum* inoculum in the soil. This result should alert growers of the influence of the climate change on the sanitary status of the asparagus cultures. The effects of some other factors such as water activity remain to be elucidated. We also established a relationship between three crop factors and the disease index values in the field which can be used to monitor fields and will allow farmers to make certain management decisions for new plantations.

## Figures and Tables

**Table 1:** Samples codes, origin, climate, varieties, cultivation type and sowing date.

Sample	Region	Municipality	Variety	Cultivation Type	Köppen–Geiger Climate Classification System [26]	Sowing Date
1	Navarra	Ancín	Grolim	White asparagus	Oceanic (Cfb)	2012
2	Navarra	Ancín	Grolim	White asparagus	Oceanic (Cfb)	2017
3	Navarra	Ancín	Grolim	White asparagus	Oceanic (Cfb)	2009
4	Navarra	Ancín	Grolim	White asparagus	Oceanic (Cfb)	2012
5	Navarra	Ancín	Grolim	White asparagus	Oceanic (Cfb)	2017
6	Navarra	Ancín	Fortens	White asparagus	Oceanic (Cfb)	2016
7	Navarra	Ancín	Grolim	White asparagus	Oceanic (Cfb)	2014
8	Navarra	Ancín	Grolim	White asparagus	Oceanic (Cfb)	2016
9	Navarra	Ancín	Cygnus	White asparagus	Oceanic (Cfb)	2016
10	Navarra	Larraga	Grolim	White asparagus	Oceanic (Cfb)	2009
11	Navarra	Berbinzana	Grolim	White asparagus	Oceanic (Cfb)	2009
12	Navarra	Berbinzana	Grolim	White asparagus	Oceanic (Cfb)	2009
13	Navarra	Miranda de Arga	Grolim	White asparagus	Oceanic (Cfb)	2010
14	Navarra	Miranda de Arga	Grolim	White asparagus	Oceanic (Cfb)	2012
15	Navarra	Tudela	Grolim	White asparagus	Warm-summer Mediterranean (Csb)	2006
16	Navarra	Tudela	Grolim	White asparagus	Warm-summer Mediterranean (Csb)	2014
17	Madrid	Aranjuez	Grande	Green asparagus	Cold semi-arid (BSk)	2011
18	Madrid	Aranjuez	Grande	Green asparagus	Cold semi-arid (BSk)	2017
19	Madrid	Aranjuez	Grande	Green asparagus	Cold semi-arid (BSk)	2012
20	Madrid	Aranjuez	Grande	Green asparagus	Cold semi-arid (BSk)	2011
21	Madrid	Aranjuez	Grande	Green asparagus	Cold semi-arid (BSk)	2014
22	Madrid	Aranjuez	Grande	Green asparagus	Cold semi-arid (BSk)	2010
23	Andalusia	Huétor-Tájar	Grande	Green asparagus	Hot-summer Mediterranean (Csa)	2010
24	Andalusia	Huétor-Tájar	Grande	Green asparagus	Hot-summer Mediterranean (Csa)	2012
25	Andalusia	Huétor-Tájar	Grande	Green asparagus	Hot-summer Mediterranean (Csa)	2012
26	Andalusia	Huétor-Tájar	Grande	Green asparagus	Hot-summer Mediterranean (Csa)	1995
27	Andalusia	Huétor-Tájar	Grande	Green asparagus	Hot-summer Mediterranean (Csa)	2016
28	Andalusia	Huétor-Tájar	Grande	Green asparagus	Hot-summer Mediterranean (Csa)	2015
29	Andalusia	Loja	Grande	Green asparagus	Hot-summer Mediterranean (Csa)	2013
30	Andalusia	Loja	Grande	Green asparagus	Hot-summer Mediterranean (Csa)	2014
31	Andalusia	Loja	Grande	Green asparagus	Hot-summer Mediterranean (Csa)	2011
32	Andalusia	Loja	Atlas	Green asparagus	Hot-summer Mediterranean (Csa)	2016
33	Andalusia	Loja	Grande	Green asparagus	Hot-summer Mediterranean (Csa)	2012
34	Andalusia	Loja	Grande	Green asparagus	Hot-summer Mediterranean (Csa)	2005
35	Andalusia	Loja	Grande	Green asparagus	Hot-summer Mediterranean (Csa)	2010
36	Andalusia	Loja	Grande	Green asparagus	Hot-summer Mediterranean (Csa)	2013
37	Andalusia	Loja	Placosesp	Green asparagus	Hot-summer Mediterranean (Csa)	2012
38	Andalusia	Loja	Placosesp	Green asparagus	Hot-summer Mediterranean (Csa)	2008
39	Andalusia	El Jau	Grande	Green asparagus	Hot-summer Mediterranean (Csa)	2010
40	Andalusia	El Jau	Grande	Green asparagus	Hot-summer Mediterranean (Csa)	2010
41	Andalusia	El Jau	Grande	Green asparagus	Hot-summer Mediterranean (Csa)	2010

**Table 2:** The *Fusarium* plant isolates used in this study for phylogenetic analysis and GeneBank accessions.

Origin	Species	Code	Isolate Source	Location	Genbank Accession No.		
					*EF-1* *α*	*RPB1*	*RPB2*
This Study	*F. oxysporum*	GR_FOAc	Asparagus Plant	Loja	MT305183	MT305069	MT305125
This study	*F. oxysporum*	GR_FOA5	Asparagus plant	Loja	MT305184	MT305070	MT305126
This study	*F. oxysporum*	GR_FOA8	Asparagus plant	Loja	MT305185	MT305071	MT305127
This study	*F. oxysporum*	GR_FOA13	Asparagus plant	Loja	MT305186	MT305072	MT305128
This study	*F. oxysporum*	GR_FOA14	Asparagus plant	Loja	MT305187	MT305073	MT305129
This study	*F. oxysporum*	GR_FOA21	Asparagus plant	Loja	MT305188	MT305074	MT305130
This study	*F. oxysporum*	GR_FOA125	Asparagus plant	Loja	MT305189	MT305075	MT305131
This study	*F. oxysporum*	GR_FOA128	Asparagus plant	Loja	MT305190	MT305076	MT305132
This study	*F. oxysporum*	GR_FOA141	Asparagus plant	Loja	MT305191	MT305077	MT305133
This study	*F. oxysporum*	GR_FOA164	Asparagus plant	Loja	MT305192	MT305078	MT305134
This study	*F. oxysporum*	GR_FOA168	Asparagus plant	Loja	MT305193	MT305079	MT305135
This study	*F. oxysporum*	GR_FOA179	Asparagus plant	Loja	MT305194	MT305080	MT305136
This study	*F. oxysporum*	GR_FOA230	Asparagus plant	El Jau	MT305195	MT305081	MT305137
This study	*F. oxysporum*	GR_FOA247	Asparagus plant	El Jau	MT305196	MT305082	MT305138
This study	*F. oxysporum*	GR_FOA249	Asparagus plant	El Jau	MT305197	MT305083	MT305139
This study	*F. oxysporum*	NA_FOA01	Asparagus plant	Ancín	MT568933	MT568949	MT568965
This study	*F. oxysporum*	NA_FOA04	Asparagus plant	Ancín	MT568934	MT568950	MT568966
This study	*F. oxysporum*	NA_FOA08	Asparagus plant	Ancín	MT568935	MT568951	MT568967
This study	*F. oxysporum*	NA_FOA10	Asparagus plant	Ancín	MT568936	MT568952	MT568968
This study	*F. oxysporum*	NA_FOA12	Asparagus plant	Berbinzana	MT568937	MT568953	MT568969
This study	*F. oxysporum*	NA_FOA13	Asparagus plant	Berbinzana	MT568938	MT568954	MT568970
This study	*F. oxysporum*	NA_FOA14	Asparagus plant	Berbinzana	MT568939	MT568955	MT568971
This study	*F. oxysporum*	NA_FOA15	Asparagus plant	Tudela	MT568940	MT568956	MT568972
This study	*F. oxysporum*	MA_FOA16	Asparagus plant	Aranjuez	MT568941	MT568957	MT568973
This study	*F. oxysporum*	MA_FOA17	Asparagus plant	Aranjuez	MT568942	MT568958	MT568974
This study	*F. oxysporum*	MA_FOA19	Asparagus plant	Aranjuez	MT568943	MT568959	MT568975
This study	*F. oxysporum*	MA_FOA20	Asparagus plant	Aranjuez	MT568944	MT568960	MT568976
This study	*F. oxysporum*	MA_FOA23	Asparagus plant	Aranjuez	MT568945	MT568961	MT568977
This study	*F. oxysporum*	MA_FOA24	Asparagus plant	Aranjuez	MT568946	MT568962	MT568978
This study	*F. oxysporum*	MA_FOA25	Asparagus plant	Aranjuez	MT568947	MT568963	MT568979
This study	*F. oxysporum*	MA_FOA27	Asparagus plant	Aranjuez	MT568948	MT568964	MT568980
This study	*F. proliferatum*	GR_FPb	Asparagus plant	Loja	MT305198	MT305084	MT305140
This study	*F. proliferatum*	GR_FP3	Asparagus plant	Loja	MT305199	MT305085	MT305141
This study	*F. proliferatum*	GR_FP17	Asparagus plant	Loja	MT305201	MT305086	MT305143
This study	*F. proliferatum*	GR_FP24	Asparagus plant	Loja	MT305202	MT305087	MT305144
This study	*F. proliferatum*	GR_FP86	Asparagus plant	Loja	MT305203	MT305088	MT305145
This study	*F. proliferatum*	GR_FP87	Asparagus plant	Loja	MT305204	MT305089	MT305146
This study	*F. proliferatum*	GR_FP100	Asparagus plant	Loja	MT305205	MT305090	MT305147
This study	*F. proliferatum*	GR_FP120	Asparagus plant	Loja	MT305206	MT305091	MT305148
This study	*F. proliferatum*	GR_FP130	Asparagus plant	Loja	MT305207	MT305092	MT305149
This study	*F. proliferatum*	GR_FP172	Asparagus plant	Loja	MT305208	MT305093	MT305150
This study	*F. proliferatum*	GR_FP182	Asparagus plant	Loja	MT305210	MT305094	MT305152
This study	*F. proliferatum*	GR_FP234	Asparagus plant	El Jau	MT305211	MT305095	MT305153
This study	*F. proliferatum*	GR_FP248	Asparagus plant	El Jau	MT305212	MT305096	MT305154
This study	*F. proliferatum*	NA_FPA01	Asparagus plant	Larraga	MW091265	MW091281	MW091299
This study	*F. proliferatum*	NA_FPA02	Asparagus plant	Ancín	MW091266	MW091282	MW091300
This study	*F. proliferatum*	MA_FPA04	Asparagus plant	Aranjuez	MW091267	MW091284	MW091302
This study	*F. proliferatum*	MA_FPA06	Asparagus plant	Aranjuez	MW091268	MW091286	MW091304
This study	*F. proliferatum*	MA_FPA08	Asparagus plant	Aranjuez	MW091269	MW091288	MW091306
This study	*F. proliferatum*	MA_FPA10	Asparagus plant	Aranjuez	MW091270	MW091290	MW091308
This study	*F. proliferatum*	MA_FPA11	Asparagus plant	Aranjuez	MW091271	MW091291	MW091309
This study	*F. proliferatum*	MA_FPA12	Asparagus plant	Aranjuez	MW091272	MW091292	MW091310
This study	*F. proliferatum*	MA_FPA15	Asparagus plant	Aranjuez	MW091275	MW091293	MW091313
This study	*F. proliferatum*	MA_FPA16	Asparagus plant	Aranjuez	MW091276	MW091294	MW091314
This study	*F. redolens*	GR_FRa	Asparagus plant	Loja	MT305213	MT305097	MT305155
This study	*F. redolens*	GR_FR4	Asparagus plant	Loja	MT305214	MT305098	MT305156
This study	*F. redolens*	GR_FR7	Asparagus plant	Loja	MT305215	MT305099	MT305157
This study	*F. redolens*	GR_FR18	Asparagus plant	Loja	MT305216	MT305100	MT305158
This study	*F. redolens*	GR_FR20	Asparagus plant	Loja	MT305217	MT305101	MT305159
This study	*F. redolens*	GR_FR23	Asparagus plant	Loja	MT305218	MT305102	MT305160
This study	*F. redolens*	GR_FR29	Asparagus plant	Loja	MT305219	MT305103	MT305161
This study	*F. redolens*	GR_FR58	Asparagus plant	Loja	MT305220	MT305104	MT305162
This study	*F. redolens*	GR_FR104	Asparagus plant	Loja	MT305221	MT305105	MT305163
This study	*F. redolens*	GR_FR157	Asparagus plant	Loja	MT305224	MT305107	MT305165
This study	*F. redolens*	GR_FR186	Asparagus plant	Loja	MT305225	MT305108	MT305166
This study	*F. redolens*	GR_FR215	Asparagus plant	El Jau	MT305226	MT305109	MT305167
This study	*F. redolens*	NA_FRA01	Asparagus plant	Ancín	MW091277	MW091295	MW091315
This study	*F. redolens*	NA_FRA02	Asparagus plant	Ancín	MW091278	MW091296	MW091316
This study	*F. redolens*	NA_FRA03	Asparagus plant	Ancín	MW091279	MW091297	MW091317
This study	*F. redolens*	NA_FRA04	Asparagus plant	Berbinzana	MW091280	MW091298	MW091318
GenBank	*F. oxysporum*	RBG5769	Soil	Australia	KJ397041	KJ397185	KJ397221
GenBank	*F. oxysporum*	RBG5796	Soil	Australia	KJ397061	KJ397205	KJ397241
GenBank	*F. oxysporum*	RBG5801	Soil	Australia	KJ397062	KJ397206	KJ397242
GenBank	*F. oxysporum*	RBG5807	Soil	Australia	KJ397066	KJ397210	KJ397246
GenBank	*F. oxysporum*	RBG5765	Soil	Australia	KJ397075	KJ397219	KJ397255
GenBank	*F. oxysporum*	RBG5771	Soil	Australia	KJ397042	KJ397186	KJ397222
GenBank	*F. oxysporum*	RBG5782	Soil	Australia	KJ397051	KJ397195	KJ397231
GenBank	*F. oxysporum*	RBG5818	Soil	Australia	KJ397072	KJ397216	KJ397252
GenBank	*F. oxysporum*	RBG5768	Soil	Australia	KJ397040	KJ397184	KJ397220
GenBank	*F. oxysporum*	RBG5779	Soil	Australia	KJ397048	KJ397192	KJ397228
GenBank	*F. oxysporum*	RBG5781	Soil	Australia	KJ397050	KJ397194	KJ397230
GenBank	*F. oxysporum*	RBG5784	Soil	Australia	KJ397053	KJ397197	KJ397233
GenBank	*F. oxysporum*	RBG5697	Soil	Australia	KJ397064	KJ397208	KJ397244
GenBank	*F. oxysporum*	RBG5783	Soil	Australia	KJ397052	KJ397196	KJ397232
GenBank	*F. oxysporum*	RBG5794	Soil	Australia	KJ397060	KJ397204	KJ397240
GenBank	*F. oxysporum*	RBG5817	Soil	Australia	KJ397071	KJ397215	KJ397251
GenBank	*F. commune*	NRRL 28387	*Dianthus caryophyllus*	Netherlands	HM057338	JX171525	JX171638
GenBank	*F. proliferatum*	NRRL 62905	*Zea mays*	USA	MN193865	MN193921	MN193893
GenBank	*F. proliferatum*	NRRL 43617	Human	USA	HM347124	HM347185	EF470206
GenBank	*F. fujikuroi*	NRRL 5538	*Saccharum officinarum*	Taiwan	MN193860	MN193916	MN193888
GenBank	*F. concentricum*	NRRL 25181	*Musa sapientum*	Costa Rica	MT010992	MT010942	MT010981
GenBank	*F. sacchari*	NRRL 66326	Lab cross	USA	MN193868	MN193924	MN193896
GenBank	*F. bulbicola*	NRRL 13618	*Nerine bowdenii*	Germany	KF466415	KF466394	KF466404
GenBank	*F. subglutinans*	NRRL 22016	*Zea mays*	USA	HM057336	JX171486	JX171599
GenBank	*F. begoniae*	NRRL 25300	*Begonia elatior*	Germany	MN193858	MN193914	MN193886
GenBank	*F. verticillioides*	NRRL 20956	*Zea mays*	USA	MN193873	MN193929	MN193901
GenBank	*F. nygamai*	NRRL 26421	Human	Egypt	HM347121	HM347147	EF470127
GenBank	*F*, *napiforme*	NRRL 25196	*Pennisetum typhoides*	South Africa	MN193863	MN193919	MN193891
GenBank	*F. oxysporum*	NRRL 26360	Human	USA	HM347120	HM347146	EF470126
GenBank	*F. redolens*	NRRL 54967	Feline	USA	KC808221	KC808300	KC808363
GenBank	*F. redolens*	NRRL 22901	*Pseudotsuga menziesii*	Canada	MT409452	MT409432	JX171616
GenBank	*F. spartum*	NRRL 66894	*Macrochloa tenacissima*	Tunisia	MT409457	MT409437	MT409447
GenBank	*F. hostae*	NRRL 29888	*Hosta* sp.	USA	MT409455	MT409435	MT409445

**Table 3:** Equations and correlation coefficients between Field Disease Index (FDI) and Colony Forming Units (CFU) per gram of soil analyzed, evaluated for every *Fusarium* species.

Adjustment	*n*	Adjusted Equation	Correlation Coefficient	*p*-Value	R^2^
Y^2^ equation	44	FDI = sqrt (256.328 − 0.184087·CFU *F. oxysporum*/g soil)	0.320118	0.0341	10.25%
Y^2^ equation	44	FDI = sqrt (858.517 − 0.211922·CFU *F. solani*/g soil)	−0.131103	0.3963	1.72%
Y^2^ equation	44	FDI = sqrt(639.741 + 0.0475964·CFU *F. proliferatum*/g soil)	0.0334676	0.8292	0.11%
Y^2^ equation	44	FDI = sqrt (709.614 − 0.29721·CFU *F. redolens*/g soil)	−0.0913385	0.5554	0.83%
Y^2^ equation	44	FDI = sqrt (411.435 + 0.121763·CFU *F. incarnatum*/g soil)	0.170733	0.2678	2.91%
Y^2^ equation	44	FDI = sqrt (571.778 + 0.19812·CFU *F. culmorum*/g soil	0.192981	0.2094	3.72%

**Table 4:** *Fusarium* spp. monoconidial isolates from symptomatic asparagus plants and soils of asparagus monoculture fields, tested for pathogenicity in pot experiments under growth-chamber conditions.

	*Fusarium* Species					
***Fusarium* sp.** **Plant Isolates**	***F. oxysporum***	***F. proliferatum***	***F. redolens***	**FSSC**		
Number of isolates	10	11	8	8		
Number of pathogenic	9	10	7	0		
Percent pathogenic	90	90.9	87.5	0		
***Fusarium* sp.** **soil isolates**	***F. oxysporum***	***F. proliferatum***	***F. equiseti***	**FSSC**	***F. compactum***	***F. acuminatum***
Number of isolates	87	14	41	68	16	32
Number of pathogenic	56	13	0	0	0	0
Percent pathogenic	64.4	92.9	0	0	0	0

**Table 5:** Mean severity on roots and percentage of frond and root weight loss of “Grande F1” asparagus plants inoculated with *Fusarium* spp. isolates grown under growth-chamber conditions.

***Fusarium* sp. Plant Isolates**	**Severity on Roots**	**Frond Weight Loss (%)**	**Root Weight Loss (%)**
*F. proliferatum* (*n* = 11)	3.0 ± 0.2 **A**	59.0 ± 3.9 **A**	82.7 ± 1.7 **A**
*F. oxysporum* (*n* = 10)	2.2 ± 0.2 **B**	57.6 ± 4.0 **A**	70.1 ± 3.5 **B**
*F. redolens* (*n* = 8)	2.4 ± 0.2 **B**	47.7 ± 4.1 **B**	65.8 ± 4.3 **B**
FSSC (*n* = 8)	0.0 ± 0.0 **C**	10.1 ± 4.9 **C**	0.0 ± 0.0 ^x^ **C**
Control (*n* = 5)	0.0 ± 0.0 **C**	0.0 ± 0.0 **C**	0.0 ± 0.0 **C**
*p*-value	***	***	***
***Fusarium* sp. Soil Isolates**	**Severity on Roots**	**Frond Weight Loss (%)**	**Root Weight Loss (%)**
*F. proliferatum* (*n* = 12)	3.5 ± 0.8 **A**	49.3 ± 14.0 **A**	71.0 ± 13.0 **A**
*F. oxysporum* (*n* = 49)	2.3 ± 1.1 **B**	20.9 ± 14.9 **B**	47.9 ± 20.3 **B**
Control (*n* = 4)	0.0 ± 0.0 **C**	0.0 ± 0.0 **C**	0.0 ± 0.0 **C**
*p*-value	***	***	***

Mean ± standard error of final scores are averaged over the number of isolates for each species. Significance through ANOVA test for weight loss (*** significance level at *p* < 0.001), all-pairwise comparisons were performed trough LSD (less significant difference) at 99.9% confidence. Significance through Kruskal–Wallis test for disease severity (*** significance level at *p* < 0.001), comparisons of mean ranks were performed at 99.9% confidence. ^x^ FSSC root weight loss was evaluated as 0, because the original value was negative, although there was not significance.

**Table 6:** Genetic diversity parameters of *Fusarium* species associated to asparagus diseased plants estimated by multilocus analysis of *EF-1α*, *RPB1*, and *RPB2* partial sequences.

*Fusarium* spp.	No. of Isolates	No. of Haplotypes	Haplotype (gene) Diversity (H)	Nucleotide Diversity (π)
*F. oxysporum*	31	14	0.888 ± 0.043	0.0044 ± 0.0004
*F. proliferatum*	23	9	0.779 ± 0.074	0.0017 ± 0.0004
*F. redolens*	16	8	0.875 ± 0.059	0.0015 ± 0.0003

## Supplementary Materials

The following tables are available online at https://www.mdpi.com/2309-608X/6/4/336/s1, Table S1: Primers used in this study for the amplification of partial sequences of the translation elongation factor-1α (*EF-1 α*), and the DNA-directed RNA polymerase II largest (*RPB1*) and second largest subunit (*RPB2*) genes of *Fusarium* spp. isolates from asparagus plants Table S2: Genetic differentiation between *Fusarium oxysporum* f. sp. *asparagi* preassigned populations by Chi square test [41].
