# Supplementary material for: Fusarium Consortium Populations Associated with Asparagus Crop in Spain and Their Role on Field Decline Syndrome
Source: J Fungi (Basel). 2020 Dec 4;6(4):336. doi: 10.3390/jof6040336 (PMC7761792; doi:10.3390/jof6040336)
Supplement: Supplementary file 1 [file jof-06-00336-s001.pdf]

Table S1. Primers used in this study for the amplification of partial sequences of the translation elongation factor-1α (EF-1α), and the DNA-directed RNA polymerase II largest (RPB1) and second largest subunit (RPB2) genes of Fusarium spp. isolates from asparagus plants.

Primer	Sequence (5'-3')	Locus	Size (bp)	Reference
ef1	ATGGGTAAGGARGACAAGAC	*EF1-α*	716	O'Donnell *et al.,*1998
ef2	GGARGTACCAGTSATCATGTT			O'Donnell *et al.*, 1998
Fa	CAYAARGARTCYATGATGGGWC	*RPB1* 5' region	1127	Hofstetter *et al.*, 2007
R8	CAATGAGACCTTCTCGACCAGC			O'Donnell *et al.*, 2010
5f2	GGGGWGAYCAGAAGAAGGC	*RBP2* 5' region	1000	Reeb *et al*, 2004
7cR	CCCATRGCTTGYTTRCCCAT			Liu *et al.*, 1999

Table S2. Genetic differentiation between *Fusarium oxysporum* f. sp. *asparagi* preassigned populations by Chi square test (Hudson et al., 1992).

Population	Chi^2	*p*	significance of *p*
Navarra-Madrid	13,333	0.1009	ns
Navarra-Andalusia	11,208	0.2617	ns
Madrid-Andalusia	17,122	0.1451	ns
Navarra-Madrid-Andalusia	38,459	0.0900	ns

ns: not significant
